# Nuclear Expression of β-Catenin Is Associated with Improved Outcomes in Endometrial Cancer

**DOI:** 10.3390/diagnostics12102401

**Published:** 2022-10-03

**Authors:** Valeria Masciullo, Tommaso Susini, Giacomo Corrado, Marina Stepanova, Alessandro Baroni, Irene Renda, Francesca Castiglione, Corrado Minimo, Alfonso Bellacosa, Benito Chiofalo, Enrico Vizza, Giovanni Scambia

**Affiliations:** 1 Division of Gynecologic Surgery, Department of Woman, Child and Public Health, Fondazione Policlinico Universitario A. Gemelli—IRCCS, Catholic University of Sacred Heart, 00168 Rome, Italy; 2 Department of Obstetrics and Gynecology, University of Florence, 50121 Florence, Italy; 3 Division of Gynecologic Oncology, Department of Woman, Child and Public Health, Fondazione Policlinico Universitario A. Gemelli—IRCCS, Catholic University of Sacred Heart, 00168 Rome, Italy; 4 Cancer Signaling and Epigenetics Program and Cancer Epigenetics Institute, Fox Chase Cancer Center, Philadelphia, PA 19111, USA; 5 Department of Pathology, University of Florence, 50121 Florence, Italy; 6 Department of Pathology and Laboratory Medicine, Albert Einstein Medical Center, Philadelphia, PA 19111, USA; 7 Gynecologic Oncology Unit, Department of Experimental Clinical Oncology, IRCCS Regina Elena National Cancer Institute, 00128 Rome, Italy

**Keywords:** β-catenin, endometrial cancer, prognosis

## Abstract

Beta-catenin is involved in intercellular adhesion and participates in the Wnt signaling pathway. This study evaluated the expression pattern and prognostic value of β-catenin in a series of endometrial carcinoma patients. Immunohistochemical analyses were used to assess the expression and subcellular localization of β-catenin from tissue sections of 74 patients with endometrial carcinoma. No correlation was found between beta-catenin expression and clinicopathological parameters. Patients expressing nuclear β-catenin (*n* = 13; 16%) showed a more favorable prognosis than patients expressing membranous β-catenin; the 5-year disease-related survival rate was 100% for cases expressing nuclear β-catenin, compared with 73.8% (SE 0.08) of cases expressing membranous β-catenin (*p* = 0.04). Although statistical significance was not reached (*p* = 0.15), cases expressing nuclear β-catenin showed a 5-year disease-free survival rate of 90.9% (SE 0.08) compared with 67.4% (SE 0.08) of cases expressing membranous β-catenin. Univariate Cox analysis revealed that membranous β-catenin expression was found to be associated with a relative risk of death of 33.9 (*p* = 0.04). The stage of disease (*p* = 0.0006), histology (*p* = 0.003), and grading (*p* = 0.008) were also significantly correlated with disease-free survival according to univariate Cox analyses. Determining β-catenin expression and localization patterns may predict survival in patients with endometrial cancer and, therefore, should be considered a potential prognostic marker of disease.

## 1. Introduction

Endometrial cancer is the fourth most common cancer in women and the 15th most common cancer overall. Uterine cancer incidence rates continue to increase by about 1% per year, and it has been estimated that there are approximately 66,500 new cases of endometrial cancer with over 12,000 deaths reported annually in the United States [1]. Multiple efforts have been made to identify molecular events that correlate with the worsening prognosis of this disease. A lack of consensus among expert pathologists in the morphologic assessment of histotype and grade is a long-standing problem. The lack of diagnostic reproducibility in our current classification affects clinical research; thus, the only way to break this impasse is to develop a robust molecular classifier as suggested by recent studies [2,3,4]. For this reason, attempts to stratify patients with endometrial cancer into different groups based on a combination of mutation and protein expression analyses have been pursued recently. A molecular classification of endometrial cancer patients may help in identifying patients who could benefit from more aggressive therapies.

β catenin is a multifunctional protein, playing a central role in development and proliferation through the regulation of E-cadherin-mediated cell adhesion and Wnt/Wingless signaling pathways [5]. In the absence of Wnt stimulation, this pathway is regulated by a multiprotein complex consisting of axin, GSK3β, and the tumor suppressor adenomatous polyposis coli (APC). GSK3β-mediated phosphorylation of the NH2-terminal β-catenin residues marks the free cytosolic protein for ubiquitination and degradation by proteasomes [6]. When Wnt ligands bind to a Frizzled-LRP (Lipoprotein receptor-related protein) cell surface receptor, they antagonize the action of GSK-3β, severely impairing the downregulation of β-catenin. Excess cytoplasmic β-catenin leads to its translocation into the nucleus where it can increase transcription of specific target genes via Lef/Tcf promoters. The Wnt pathway is abnormal in many cancers [7]. Endometrial cancer frequently has mutations in the putative GSK-3β phosphorylation sites of β-catenin [8,9,10,11]. Previous studies in endometrial cancer and its precursor lesions, have shown distinct subcellular expression patterns of β-catenin [12,13], but data regarding correlation with oncologic outcomes are inconclusive [14,15,16].

The aim of this study was to investigate the expression patterns of the cellular adhesion marker β-catenin and its prognostic significance within a well-defined population-based series of endometrial carcinoma with complete follow-ups.

## 2. Materials and Methods

This is a multicenter retrospective pilot study. The study was approved by the institutional review board of the Fondazione Policlinico Universitario “Agostino Gemelli”—IRCCS, a center promoting the study (CICOG-31-10-18/111). The design, analysis, interpretation of data, drafting, and revisions follow the Helsinki Declaration, the Committee on Publication Ethics guidelines (http://publicationethics.org/, accessed on 31 October 2018), and the reporting of studies conducted using observational, routinely collected health data statements that are available using the Enhancing the Quality and Transparency of Health Research Network (www.equator-network.org, accessed on 31 October 2018). The study was unadvertised, and no remuneration was offered to encourage patients to give consent for the collection and analysis of their data. Each patient enrolled in this study was informed about the aims and procedures and signed an informed consent to allow data collection for research purposes.

### 2.1. Population and Study Design

Specimens were retrospectively collected from patients affected by endometrial carcinoma who underwent surgery in the Division of Gynecology of the Careggi Hospital, University of Florence, Italy. Immunohistochemical analysis of tissue sections in the Department of Pathology and Laboratory Medicine, Albert Einstein Medical Center, Philadelphia, PA, was used to assess the expression and subcellular localization of β-catenin. Tumors were histologically classified as well (G1), moderately (G2), and poorly (G3) differentiated using the World Health Organization (WHO) system. Clinical stages of disease were established according to the 2009 International Federation of Gynecology and Obstetrics’s (FIGO) system. Recurrence was considered as any documented relapse of the tumor either in the pelvis or systemically.

Disease-free survival (dfs) was calculated from the date of surgery. Patients who had a residual tumor after surgery or had an early recurrence (within three months after surgery) were excluded from the disease-free analysis and considered alive with disease. Patients who died from a different cause than endometrial cancer were considered lost at follow-up; their survival time was concluded at the date of death.

### 2.2. Immunohistochemistry and Specificity of Immunostaining

After surgical resection, each tumor specimen was immediately formalin-fixed and then paraffin-embedded for routine and immunohistochemical investigation. For immunohistochemistry staining, 3 μm-thick sections were taken on poly-l-Lysine coated slides. Briefly, the polyclonal antibody to β catenin (sc-1496) (Santa Cruz Biotechnology, Santa Cruz, CA, USA) was incubated overnight with tissue sections according to the kit’s instructions. The peptide was used to generate preabsorption to assess the staining specificity of β-catenin. The pattern of staining observed with the polyclonal antibody was confirmed on duplicate slides using a monoclonal antibody (sc-7963) (Santa Cruz Biotechnology, Santa Cruz, CA, USA). For the negative control, the primary antibody was substituted for PBS. 

### 2.3. β Catenin Scoring

There were two patterns of β-catenin expression described: membranous, if β-catenin was only expressed in the cell membrane; and nuclear, when β-catenin was localized in the nucleus, irrespective of the percentage of stained nuclei or simultaneous expression of β-catenin in the membrane and cytoplasm. As previously discussed, membranous β-catenin expression was estimated semi-quantitatively and subjectively (C.M., FC) [17]. At least 20 high-power fields were chosen randomly, and 2000 cells were counted. Every tumor and synchronous endometrial lesion was assessed and given a score, obtained by multiplying the intensity of the staining (no staining = 0; low level = 1; medium staining = 2; strong staining = 3) by the percentage of cells stained (0% = 0; under 10% = 1; 10–50% = 2; 51–80% = 3; over 80% = 4). The maximum score is 12 with this system. In subsequent statistical analyses, cutoff points for score categories were mainly based on median or quartile values, with the frequency distribution of β-catenin also considered.

### 2.4. DNA Analysis

DNA was extracted from paraffin blocks containing a large area of epithelial tumor cells (>75%) expressing nuclear β-catenin using the QIAamp DNA FFPE tissues kit (56404, Quiagen, Valencia, CA, USA). The primers forward 5′-GCTGATTTGATGGAGTTGG-3′ and reverse 3′-CTCTTACCAGCTACTTGTTC-5′ were used to amplify DNA sequences of the third coding exon of the β1-catenin gene (MS) [18]. A reaction mixture containing 20–100 ng of genomic DNA, 10 pmol of each primer, 250 mM of each dNTP, 10× PCR buffer (Perkin-Elmer Applied Biosystems Division, Foster City, CA, USA), and 0.5 units of AmpliTaq Gold (Perkin-Elmer) was used for the PCR. Initial DNA denaturation was obtained by heating the mixture at 95 °C for 5 min, followed by 30 cycles of denaturation (at 95 °C for 1 min), annealing (at 51 °C for 1 min), and extension (at 72 °C for 1 min) on the PTC-200 (MJ Research). Sanger sequencing of PCR products was performed using BigDye v3.1 (Applied Biosystems, Foster City, CA, USA). After that, the same primers were used for amplification, and sequencing reactions were run on an ABI Prism 310 Genetic Analyzer (Applied Biosystems). 

### 2.5. Statistical Analysis

The χ^2^ and the Fisher’s exact test were used to analyze the distribution of β-catenin-positive specimens according to clinicopathological characteristics. Disease-free survival (dfs) and disease-related survival (drs) were calculated according to the Kaplan–Meier method and evaluated by the log-rank test. To evaluate the effect of each prognostic variable on dfs and drs, Univariate Cox analyses were used. Finally, a multivariate analysis (Cox proportional-hazards regression) was used to estimate which possible risk factors yielded independent prognostic information. The reported *p* values are two-sided, and *p* values of less than 0.05 were considered to indicate statistical significance. Data analyses were carried out using SPSS Statistical Software, release 5.0.1 (SPSS Inc., Chicago, IL, USA).

## 3. Results

Eighty-one specimens were retrospectively collected from patients affected by endometrial carcinoma; however, clinicopathological parameters and follow-up were available in only 74 cases. Patients’ ages ranged from 46 to 84 years, with a median age of 64 years. 

Neither radiotherapy nor chemotherapy were received by patients before surgery.

Adjuvant radiotherapy was administered to the whole pelvis (56 Gy) after surgery in patients with codified risk factors.

Adjuvant chemotherapy with a standard regimen including carboplatin and paclitaxel every 21 days, for six cycles, was administered to those patients with a more advanced stage of the disease.

After adjuvant treatments, patients were evaluated every three months for the first two years, every four months during the third and fourth years, and every six months thereafter.

### 3.1. β-Catenin Protein Expression in Normal Human Endometrium, Simple and Complex Hyperplasia and Cystic Atrophy

The expression of β-catenin in endometrial tissues was evaluated using immunohistochemical analysis. Lesions synchronous with invasive carcinomas were classified as normal human endometrium (*n* = 12), simple and complex hyperplasia (*n* = 7), and atrophy (*n* = 3). Normal endometrium adjacent to the adenocarcinoma showed a high percentage of β-catenin staining in the examined specimens (mean 96%, median 100%, and range 50–100%), whereas simple and complex hyperplasia showed decreased percentages of β-catenin-stained cells (mean 75%, median 75%, range 40–100%). In cystic atrophy, β-catenin staining also decreased (mean 47%, median 40%, range 20–80%). β-catenin immunostaining was mostly membranous, but weakly cytoplasmic staining was also observed. 

### 3.2. β-Catenin Expression in Endometrial Adenocarcinomas and Correlations with Clinicopathological Features

**Figure 1:**
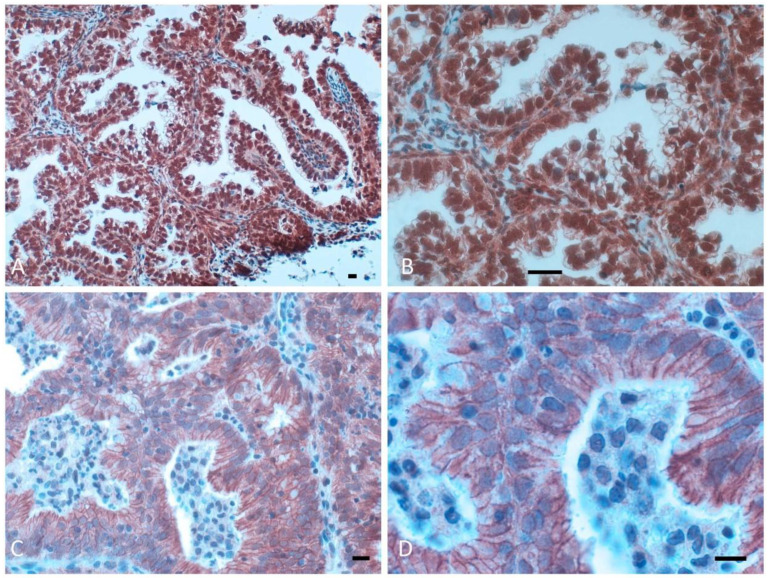
Different patterns of β-catenin expression in endometrial cancer: strong nuclear expression in (**A**) (×200) and (**B**) (×400); membranous expression absent in (**C**) (×400) or weak in (**D**) (×600) nuclear expression of β-catenin. Scale bar = 50 μm.

All analyzed tumors had β-catenin expression in the cellular membrane, with variable extension and intensity, whereas simultaneous nuclear β-catenin expression (nuclear pattern) was observed in only 13 (16%) endometrial carcinomas (Figure 1A,B); the remaining 68 tumors (84%) only had membrane expression (membranous pattern) (Figure 1C,D). The percentage of tumor cells expressing nuclear β-catenin varied from 3% to 100% and was frequently found in areas with squamous differentiation. The expression pattern (nuclear versus membranous) did not correlate significantly with any of the clinico-pathological parameters examined (Table 1). However, we did observe a trend toward a relationship between nuclear expression (8 out of 12 cases) and a high grade of differentiation (G1) (*p* = 0.08). Post-operative therapy was also similar in tumors with a nuclear or membranous β-catenin expression pattern. As previously reported, only 14 of the 74 carcinomas with β-catenin expression in the membrane had preserved membrane expression (19%); the remaining 60 carcinomas had reduced membrane expression (81%). An investigation was performed on distribution of β-catenin according to a series of clinicopathological parameters (age, FIGO stage, grading, histotype, depth of myometrial invasion, adjuvant treatment, ploidy status, S phase, DNA index, and pRb2/p130 and p27 expression) obtained from all 74 patients enrolled (Table 2). There was no correlation between the immunoreactivity expression of β catenin (preserved versus reduced) and other clinicopathological parameters.

### 3.3. β-Catenin Gene Mutations

No β-catenin gene mutations were observed in all endometrial cancers (*n* = 13) with nuclear β catenin expression.

### 3.4. Correlation of β-Catenin Expression with Clinicopathological Parameters and Survival Analysis

**Figure 2:**
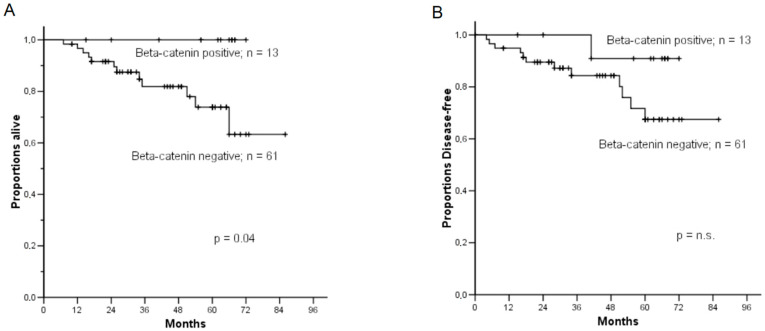
Kaplan–Meier plot of disease-related survival (**A**) and disease-free survival (**B**) according to β-catenin expression patterns (nuclear vs. membranous). The 5-year disease-related survival (drs) rate was 100% for specimens expressing nuclear β-catenin, compared with 73.8% of specimens expressing membranous β-catenin (*p* ≤ 0.04), whereas cases expressing nuclear β-catenin showed a 5-year disease-free survival (dfs) rate of 90.9% compared with 67.4% of cases expressing membranous β-catenin, although the statistical significance was not reached (*p* = 0.15).

Data on oncological follow-up were available for 74 patients (median follow-up, 47 months; range 10–86 months). Progression and death from the disease were observed, respectively, in 13 and 12 patients. A more favorable prognosis was observed in nuclear β-catenin expression patterns than the membranous β-catenin expression pattern specimens; the 5-year disease-related survival (drs) rate was 100% for patients expressing nuclear β-catenin, compared with 73.8% (SE 0.08) of patients expressing membranous β-catenin (*p* = 0.04) (Figure 2A). Even if the statistical significance was not reached due to the small number of patients with a positive beta-catenin (*p* = 0.15), the 5-year disease-free survival (dfs) rate was 90.9% (SE 0.08) for cases expressing nuclear β-catenin compared with 67.4% (SE 0.08) of cases expressing membranous β-catenin (Figure 2B). For both drs and dfs, the prognostic role of age at diagnosis, stage, histological grade, depth of myometrial invasion, and β-catenin expression status were tested using univariate Cox analyses (Table 3). Membranous β-catenin expression was found to be associated with a relative risk (RR) of dying at 33.9 (*p* = 0.04). Univariate Cox analyses also showed the stage of disease (*p* = 0.02) and grading (*p* = 0.01) to be significantly associated with disease-related survival (Table 3). The stage of disease (*p* = 0.0006), histology (*p* = 0.003), and grading (*p* = 0.008) were also significantly correlated with disease-free survival according to univariate Cox analyses (Table 3).

In a multivariate analysis for disease-free survival, the stage of disease only retained an independent prognostic role with a hazard ratio of 5.89 (95% CI 1.29–26.75; *p* = 0.02).

## 4. Discussion 

Currently, the integration of molecular risk factors with clinicopathological parameters in endometrial cancer leads to improved risk stratifications with potential clinical utility [19,20]. This integrated molecular risk prediction promises to innovate endometrial cancer staging and treatment based on results from prospective clinical trials. 

Beta-catenin is a transmembrane protein with many functions and plays an essential role both in cell adhesion mediated by E-cadherin and in Wnt-mediated transcriptional activation. In quiescent cells, β-catenin is rapidly degraded through a phosphorylation mechanism involving the glycogen complex synthase kinase (GSK-3β-Axin-APC). When β-catenin accumulates in the cytoplasm because it escaped the degradation mechanism, it translocates to the nucleus where the protein interacts with transcriptional factors Tcf/LEF, activating the expression of various genes such as cyclin D1 and c-myc [21,22]. β-catenin, which was originally isolated for its association with the cytoplasmic domain of cadherins (cell adhesion molecules), therefore plays a significant role in the Wnt pathway for the control of cell proliferation and death [5]. 

Mutations of β-catenin have been reported in a large variety of human cancers such as colorectal cancer, gastric cancer, hepatocarcinoma [23], malignant melanoma [24], desmoid tumors [25], craniopharyngioma, and pilomatrixoma [26]. They are considered the result of nuclear accumulation of β-catenin with the subsequent transcriptional activation of Tcf/LEF and the expression of genes involved in tumorigenesis.

Several studies on β-catenin expression in endometrial carcinoma have been published [27,28,29]. In our series of 74 endometrial carcinomas, nuclear β-catenin staining was found in 13 of 74 tumors (18%), which is consistent with the rate reported in other studies [16,27]. We observed β-catenin expression in 75% in endometrioid and 25% in non-endometrioid histotypes, as previously shown [12,17,19] although the differences between the groups were not significant. A systematic review of fifteen observational studies with 1158 endometrial carcinomas showed that the nuclear expression of β-catenin is an accurate immunohistochemical surrogate of *CTNNB1* exon 3 mutations and thus might be considered in the endometrial cancer risk stratification [30]. However, as previously observed in breast cancer [31], the expression of nuclear β-catenin occurs in the absence of *CTNNB1* mutations in our 13 cases. Since several other proteins, including S100P, are regulatory components of a novel ubiquitinylation complex involved in β-catenin degradation, further studies are in need with specific regard to trigger for β-catenin nuclear localization in endometrial cancer in the absence of CTNNB1 mutations [32].

A few studies have investigated the association between β-catenin expression and clinicopathological features of EC. A loss of β-catenin expression has been reported to be associated with advanced stages and lymph node involvement [17]. As previously shown, in our series of EC, there was no correlation between β-catenin expression and clinicopathological parameters [19]. This difference may result from methodological bias and the use of different antibodies.

A TCGA analysis focused on 271 endometrioid carcinomas of the endometrium revealed that the activation of the Wnt/β-catenin pathway in the tumor and the consequent nuclear β-catenin staining pattern had a lower overall survival of [33]. Indeed, Kurnit KC et al. showed that *CTNNB1* and *TP53* mutations were associated with worse recurrence-free survival at multivariate analyses in 342 patients with grade 1–2, stage I–II endometrioid endometrial cancer [11]. However, the clinical and prognostic value of β-catenin as a prognostic factor in EC is still controversial [12,17,34].

In our study, nuclear β-catenin expression-pattern specimens showed a more favorable prognosis than membranous β-catenin expression pattern specimens; the 5-year disease-related survival (drs) rate was 100% for specimens expressing nuclear β-catenin, compared with 73.8% of specimens expressing membranous β-catenin (*p* = 0.04). Although β-catenin nuclear expression has already been reported to identify a subset of patients with a favorable outcome in endometrioid ovarian carcinomas [35], to the best of our knowledge, this is the first time that this event is observed in EC. The fact that nuclear β-catenin expression is a molecular feature of type I endometrial cancer and mostly occurs in the early stage, low-grade EC [12] may account for this result. However, a larger number of cases involving nuclear localization due to genetic mutations are necessary to confirm this finding. 

We also observed for the first time that membranous β-catenin expression is associated with a relative risk (RR) of dying of 33.9 (*p* = 0.04). This could be because nuclear β-catenin staining is a feature of low-grade ECs, whereas membranous staining is more frequent in serous carcinomas and high-grade tumors [36]. 

## 5. Conclusions

With the increasing clinical availability of molecular tests, including molecular profiles along with the usual pathology and clinical data, the stratification of patients who may benefit from different therapies is becoming a realistic goal. Our current data suggest that β-catenin may have a different prognostic role in endometrial cancer based on its cellular localization. Prospective clinical trials on a broader number of cases are needed to confirm our hypothesis, with the ultimate goal of incorporating molecular information into routine endometrial cancer treatment algorithms.

## Figures and Tables

**Table 1:** Relationship between β-Catenin Expression and Clinicopathological Features in Endometrial Carcinomas.

	Total *n*.	Nuclear Pattern	Membranous Pattern	*p*
Age	74			
≤65 year (44)		6 (8%)	38 (51%)	N.S.
>65 year (30)		7 (10%)	23 (31%)	
FIGO stage	74			
I (49)		10 (13%)	39 (53%)	N.S.
II (14)		1 (1.4%)	13 (17.5%)	
III (8)		1 (1.4%)	7 (9.5%)	
IV (3)		1 (1.4%)	2 (2.8%)	
Histological type	74	13	61	
Adenocarcinoma endom (55)		8 (10.8%)	47 (63.5%)	N.S.
Adenosquamous (13)		4 (5.5%)	9 (12.2%)	
Clear cell (4)		1 (1.4%)	3 (4%)	
Adenoacanthoma (2)		0	2 (2.6%)	
Grading	74			
G1 (35)		8 (10.9%)	27 (36.4%)	0.08
G2 (20)		1 (1.4%)	19 (25.8%)	
G3 (19)		4 (5.4%)	15 (20.1%)	
Myometrial invasion	74			
<50% of depth (32)		6 (8.1%)	26 (35.1%)	N.S.
>50% of depth (42)		7 (9.5%)	35 (47.3%)	
Adjuvant treatment	74			
None (42)		6 (8.1%)	36 (48.7%)	N.S.
Radiotherapy (27)		6 (8.1%)	21 (28.3%)	
Chemotherapy (5)		1 (1.4%)	4 (5.4%)	

N.S., not significant.

**Table 2:** Distribution of β-catenin membrane expression levels according to patient characteristics.

	Total *n*.	Reduced β-Catenin	Preserved β-Catenin	*p*
Age	74			
≤65 year (44)		35 (47.3%)	10 (13.5%)	N.S.
>65 year (29)		25 (34%)	4 (5.2%)	
FIGO stage	74			
I (49)		39 (53%)	11 (15%)	N.S.
II (14)		11 (15%)	3 (4%)	
III (8)		7 (9.5%)	0	
IV (3)		3 (4%)	0	
Histological type	74			
Adenocarcinoma endom (69)		54 (74%)	14 (19%)	N.S.
Other hystotypes (5)		5 (7%)	0	
Grading	74			
G1 (35)		25 (34%)	10 (13.5%)	0.036
G2 (20)		16 (21%)	4 (6.5%)	
G3 (19)		18 (24%)	--	
Myometrial invasion	74			
<50% of depth (32)		24 (32%)	8 (11%)	N.S.
>50% of depth (42)		36 (49%)	6 (8%)	
Adjuvant treatment	74			
None (42)		7 (10%)	35 (47%)	N.S.
Radiotherapy (27)		5 (8%)	22 (30%)	
Chemotherapy (5)		2 (3.5%)	3 (1.5%)	

N.S., not significant.

**Table 3:** Significant predictors of clinical outcome in 74 patients with endometrial adenocarcinoma, according to the Cox Univariate Analysis for disease-related (drs) and disease-free survival (dfs).

Variable	RR of Death	95% CI	*p* Value	RR of Recurrence	95% CI	*p* Value
β catenin expression						
nuclear	1			1		
membranous	33.9	1.11–771.34	0.04	3.95	0.5–30.62	n.s.
FIGO stage						
I	1			1		
>I	3.87	1.18–12.66	0.02	4.91	1.56–15.42	0.0006
Age						
≤65 year	1			1		
>65 year	1.81	0.58–5.67	n.s	1.56	0.52–4.69	N.S.
Histotype						
Not Aggressive	1			1		
Aggressive	2.24	0.28–17.78	n.s.	5.65	1.15–27.73	0.003
Grading						
1	1			1		
2–3	14.5	1.83–115.39	0.01	7.89	1.71–36.45	0.008
Myometrial Invasion.						
<50% of depth	1			1		
>50% of depth	1.80	0.53–6.07	n.s.	2.25	0.68–7.41	N.S.

N.S., not significant.

## Data Availability

The data that support the findings of this study are available from the corresponding author upon reasonable request.
